# Presenteeism and associated factors among railway train drivers

**DOI:** 10.12688/f1000research.111999.1

**Published:** 2022-04-28

**Authors:** Asmaa El-Sayed Awaad, Sohair El-Bestar, Abdel-Hady El-Gilany, Adel Al-Wehedy, Samah Saleh El-Hadidy

**Affiliations:** 1 Assistant lecturer of Industrial Medicine and Occupational Health, Public Health & Community Medicine Department, Faculty of Medicine, Mansoura University, Mansoura, Dakahlia Governorate, 35516, Egypt; 2 Professor of Industrial Medicine and Occupational Health, Public Health & Community Medicine Department, Faculty of Medicine, Mansoura University, Mansoura, Dakahlia Governorate, 35516, Egypt; 3 Professor of Public Health and Preventive Medicine, Public Health & Community Medicine Department, Faculty of Medicine, Mansoura University, Mansoura, Dakahlia Governorate, 35516, Egypt; 4 Assistant professor of Industrial Medicine and Occupational Health, Public Health & Community Medicine Department, Faculty of Medicine, Mansoura University, Mansoura, Dakahlia Governorate, 35516, Egypt

**Keywords:** Railway, Egyptian train drivers, Presenteeism, Stanford presenteeism scale-6, Psychological distress

## Abstract

**Background:** Presenteeism is an emerging work-related health problem among train drivers. It is more serious than absenteeism, as it accounts for higher productivity losses and may increase the risk of occupational accidents. Train drivers have high rates of mental and physical health conditions that may put them at high risk of presenteeism.

**Methods:** A comparative cross-sectional study was conducted on 100 train drivers working in Mansoura railway station and 100 administrative employees working in the Faculty of Medicine, Mansoura university as a comparison group to estimate the prevalence of presenteeism and its associated factors among train drivers working in Mansoura railway station, Egypt. A questionnaire was used to collect socio-demographic, occupational and medical data. The Kessler Psychological Distress Scale (K10) was used to measure non-specific psychological distress. The Stanford Presenteeism Scale (SPS-6) was used to assess productivity loss related to sickness presenteeism.

**Results:** The prevalence of presenteeism was significantly higher among train drivers (76%) compared to the comparison group (31%). All participants (100%) with psychological distress reported presenteeism. Being a train driver (adjusted odds ratio [AOR]=5.4) and having hypertension (AOR=4.03) are independent predictors for presenteeism.

**Conclusions:** The prevalence of presenteeism and its associated risk factors were significantly higher among train drivers than the comparison group. There is an urgent need for the railway industry to understand the factors that may contribute to presenteeism.

## Introduction

Presenteeism is defined as “the phenomenon of people, despite complaints and ill health that should prompt rest and absence from work, still turning up at their jobs”. ^1^ Presenteeism can negatively affect productivity in a way similar to absenteeism. However, presenteeism involves higher productivity losses than absenteeism. ^2^ It can decrease work productivity and working safely and may increase the risk of occupational accidents. ^3^ Presenteeism is more costly to employers than absenteeism, since employers pay the employees for their attendance at work and prolonged work time to complete a task. They also pay them for any compensation as a result of errors done by sick employees. ^4^

Certain difficulties in work can increase the risk of presenteeism such as; the fear of un employment or losing a job, staff replacement, and financial difficulties. ^1^^,^^5^ Lack of control over tasks and inadequate support from coworkers are also risk factors associated with higher risk of presenteeism. ^6^^,^^7^

Presenteeism has been linked to stress at work. ^8^ Stress and subsequently psychological distress are considered to be significant contributors to presenteeism. ^9^^–^^11^

In the railway industry, presenteeism is a common problem among train drivers as they have to deal with a high level of job demands and responsibilities. ^12^ Train driving is a high-strain job which needs complex skills. A healthy physical and mental condition of train drivers is also very important since, their vigilance and attention are crucial to their job. The work-place environment and its different hazards increase the work load among train drivers. ^13^ They are exposed to several psychosocial risk factors such as; working in shifts, lone working and irregular working hours, long hours of duty with rigid protocols and little options for taking rest. These factors are considered a source of stress and mental suffering among train drivers affecting their health, interfering with their attention and concentration and directly influence the prevalence of presenteeism. ^14^^–^^16^

The presence of chronic conditions is also a major risk factor for presenteeism. Chronic conditions can lead to inadequate work performance as a result of poor physical and psychological well-being of workers. ^17^^,^^18^ Cardiovascular risk factors such as obesity, hypertension and dyslipidemia were the most prevalent health conditions in train drivers and also have the greatest impact on their fitness for duty. ^19^ Several studies have found that health risks such as obesity, physical inactivity, poor diet, hypertension, hypercholesterolemia and musculoskeletal pain are directly related to productivity loss if the workers continue to work while ill. ^10^^,^^20^ Furthermore, presenteeism may exacerbate the existing health conditions and impair the quality of life of sick employees. ^20^ To the best of the authors’ knowledge, this work is the first attempt to study the problem of presenteeism and its association with physical and psychological health status among railway train drivers in Egypt.

### Aim of work

This study aims to estimate the prevalence of presenteeism and determine its possible associated factors among train drivers working at Mansoura railway station.

## Methods

### Ethical consideration

This manuscript is abstracted from an MD thesis (not published yet) that was approved by Institutional Research Board (IRB) of Faculty of Medicine, Mansoura University, code number: MD.18.12.108. Approval of all responsible authorities was obtained. Written informed consent was obtained from participants with assurance of confidentiality. The questionnaire was anonymous. A copy from laboratory investigation was given to each participant with suitable advice in case of abnormal findings for further management.

### Study locality and duration

The study was conducted on train drivers working at Mansoura railway station located in the Central Delta Region of the Egyptian National Railways (ENR) during the period from February to November 2019.

### Study design

Cross-sectional comparative study.

### Study population

This study included all train drivers and their assistants working in Mansoura railway station. They were requested personally by the investigator and asked to participate voluntarily in the study. All eligible train drivers and their assistants who accepted to participate in the study and had the following inclusion criteria were recruited; working for at least one year, in both day and night shifts and in both passenger and freight (goods) transport. Train drivers who were away from train driving and shifted to administrative work due to medical or non-medical causes were not included in the study. The total workforce in the group was 100, including; 58 train drivers and 42 train driver assistants. An equal number of 100 participants were chosen from the administrative staff working in the Faculty of Medicine, Mansoura university as a comparison group. Both groups were matched in their socio-demographic characteristics.

### Flow of work

Train drivers were interviewed and examined at 8 am in the off days or either before or after shift in a specific room at a nearby hospital (Mogamaa Al-Eyman Hospital), near to Mansoura railway station; while for the comparison group, the study was carried out at Public Health and Community Medicine Department, Faculty of Medicine, Mansoura university, during the work day. The interview and examination of study participants were carried out in the same session personally by the investigator and lasted for 20-30 minutes for each participant, including filling in the questionnaire; clinical examination and withdrawing blood samples for laboratory investigation. The blood samples were collected between 8-9 am in a sitting position after 10-12 hours of fasting to assess lipid profile then the samples were transferred in an icebox to the laboratory of Clinical Pathology Department, Faculty of Medicine where biochemical evaluation was carried out. The research work was carried out 2-3 times weekly, at times suitable to the study participants, with an average of 8-10 participants per setting.

### Study tools

Participants in both groups were subjected to:

1) A pre-designed questionnaire to collect the following data; sociodemographic characteristics and personal history, occupational history and physical complaints in the last 12 months. ^53^ No preliminary testing was done as sociodemographics, occupational and clinical data have no scoring to create latent variable. Physical complaints were arranged according to international classification of diseases ICD-10, World health Organization version 2016 ^21^ and included; ocular, auditory, respiratory, dermatological, musculoskeletal, cardiovascular and neurological complaints. Cardiovascular complaints included; chest pain, chest tightness, shortness of breath and palpitation. Neurological complaints included; difficulty in concentration, tingling and/or numbness in toes or fingers and pain and/or weakness in distal muscles.
2) **Kessler Psychological Distress Scale (K10):** a widely-known measure of non-specific psychological distress based on behavioral, emotional, cognitive, and psychophysiological manifestations. ^22^ The questionnaire (K10) measures the frequency with which the individual developed anxiety and depression symptoms in the past month. The questionnaire consists of 10 questions, each of which has five possible response choices ranging from “none of the time” to “all of the time” with scores from 1 to 5. The highest score is 50, which indicate severe distress, and the lowest score is 10 indicating no distress. Scores of 11-19 indicates low level of distress, 20–24 indicates mild level of distress, 25–29 moderate level of distress and scores of 30–50 indicates severe or very high psychological distress. Psychological distress is determined with a score higher than 19 (>19). ^23^ Arabic translation of questions was derived from the Arabic version of the ten-item version of Kessler Psychological Distress Scale (K10). ^24^
3) **Stanford Presenteeism Scale (SPS-6):** a well-known measure of productivity loss related to sickness presenteeism. It has two parts; in the first part, prevalence of sickness presenteeism is determined using the following question; *‘During the last month have you shown up for work despite feeling sick or having a health problem that prevented you from carrying out your tasks in a normal manner?’.* ^25^ If presenteeism is detected in the first part, the second part of the questionnaire should be completed, it consists of six-items with a five-point scale of responses ranging from strongly disagree to strongly agree and scored from 1 to 5 translated in to Arabic. Questions 1, 3 and 4 evaluate the ability of the respondents to concentrate during work performance; while the questions 2, 5 and 6 assess the interference of the reported health problems with the ability to complete work. ^26^ Scores can range from 6-30, with lower scores (≤18) indicting presenteeism (decreased productivity and below-normal work quality due to an illness), and higher scores (>18) indicating a greater ability to concentrate on and accomplish work despite health problem(s). ^26^
4) **Blood pressure measurement:** a mercury sphygmomanometer (Alpk2 300-V, Japan) was used to measure blood pressure on the right arm supported at heart level in the seated position after five minutes of rest. It was measured twice at five-minute intervals and the average of both readings was used to estimate the individual’s blood pressure. ^27^ Hypertension is considered when systolic blood pressure (SBP) ≥140 mmHg and/or diastolic blood pressure (DBP) ≥90 mmHg; or current use of antihypertensive treatment. ^28^
5) **Anthropometric measurements:** body weight was measured in kilograms with a portable mechanical weighing scale (Laica LC02/e-11/2013, China). Height was measured in centimeters. Body mass index: was derived by dividing the weight in kilograms by the square of the height in meters (kg/m2). According to BMI, the subject is classified as: underweight (BMI <18.5) normal weight (BMI ≥ 18.5 to < 25), overweight (BMI ≥ 25 to < 30) and obese (BMI ≥ 30). ^29^^,^^30^
6) **Laboratory investigation:** a 2 ml peripheral blood sample was obtained from the antecubital vein of each participant after 10-12 hours of fasting for biochemical testing (lipid profile). Blood samples were collected in glass tubes and transferred immediately to the laboratory of Clinical Pathology Department, Faculty of Medicine where total cholesterol, low-density lipoprotein (LDL-cholesterol), high-density lipoprotein (HDL-cholesterol), and triglycerides serum levels were measured (Roche copus c111 analyzer, Switzerland). Dyslipidemia was defined as abnormalities in the plasma lipids occurring either singly or in combinations measured in milligrams (mg) per deciliter (dl) of blood and converted in to SI units (mmol/L), including; total cholesterol ≥200 mg/dL (≥5.18 mmol/L), LDL ≥130 mg/dl (≥3.36 mmol/L), triglycerides ≥150 mg/dl (≥1.69 mmol/L) and HDL <40 mg/dl (<1.03 mmol/L) and/or using lipid-lowering medications. ^31^^,^^32^

### Statistical analysis

Data were entered and statistically analyzed using (SPSS version 16.0, RRID:SCR_016479). Qualitative variables were expressed as numbers and percentages. Chi-square test (χ ^2^) was used for significance testing of categorical data; as appropriate. Crude odds ratios (COR) and their 95% confidence intervals (CI) were calculated. Quantitative data were described as means ±SD (standard deviation) after testing for normality using Shapiro test and for comparison between groups, independent sample t-test was used. Significant predictors of presenteeism in bivariate analysis were entered into binary stepwise logistic regression for prediction of independent predictors of presenteeism. Adjusted odds ratios (AOR) and their 95% confidence intervals (CI) were calculated. A statistically significant difference was considered at P value ≤0.05.

## Results

The number of participants at each stage of the study was 200 (100 train drivers and 100 comparison group) except for the stage of laboratory investigation where the number of participants was 185 (92 train drivers and 93 comparison group) indicating those who accepted to give blood sample for laboratory investigation. ^52^

Table 1 reveals that train drivers matched the comparison group in all sociodemographic characteristics with no statistically significant differences (P>0.05). There is no statistically significant difference (P>0.05) between duration of employment in both groups. However, the mean working hours per week is statistically significantly higher (P≤0.001) among train drivers (65.52±8.7 hours) compared to the comparison group (35.88±0.8 hours). Most of the train drivers (72%) worked alternating day and night shifts while all the comparison group (100%) worked only day shifts with a highly statistically significant difference (P≤0.001).

**Table 1.** Socio-demographic characteristics and occupational profile of the study groups.

Characteristics	Train drivers n=100	Comparison group n=100	Test of significance and P- value
	N (%)	N (%)	
**Age in years**			
**Mean ±SD**	40.8±8.78	41.5±8.45	t=0.542, P=0.6
<40	44(44)	43(43)	**χ** ^**2**^=0.02 P=0.9
≥40	56(56)	57(57)	
**Marital status**			
Single	6(6)	8(8)	**χ** ^**2**^=0.32 P=0.6
Married	94(94)	92(92)	
**Residence**			
Urban	40(40)	35(35)	**χ** ^**2**^ =0.53 P=0.5
Rural	60(60)	65(65)	
**Education level**			
Primary & preparatory	11(11)	8(8)	**χ** ^**2**^=1.73 P=0.4
Secondary (general & technical)	71(71)	67(67)	
Intermediate institute or higher	18(18)	25(25)	
**Duration of employment (years)**			
**Mean ±SD**	17.4±9.88	16.7±8.34	t=0.54, P=0.6
**Working hours per week**			
**Mean ±SD**	65.52±8.7	35.88±0.8	t=34.02, P≤ 0.001
**Type of shift** ^**a**^			
Day	15(15)	100(100)	χ ^2^=147.8 P≤0.001
Night	13(13)	0	
Alternating day and night	72(72)	0	

^a^ Day shift (6:00 a.m. to 6:00 p.m.); and night shift (6:00 p.m. to 6:00 a.m.). SD=standard deviation.

Table 2 shows that the most frequent physical complaints among train drivers during the last 12 months were musculoskeletal complaints (60%), followed by neurological (47%), then cardiovascular complaints (33%). In the comparison group, the musculoskeletal complaints (36%) ranked the first, followed by ocular (17%) and neurological complaints (17%). Almost all physical complaints were more frequent among train drivers compared to the comparison group with a statistically significant difference (P≤0.05) except for auditory and dermatological complaints where the difference was statistically not significant (P>0.05)

**Table 2.** Distribution of physical complaints of the study groups in the past 12 months.

Physical complaints	Train drivers n=100	Comparison Group n=100	Test of significance and P value
	N (%)	N (%)	
**Ocular**	30(30)	17(17)	χ ^2^=4.7 P=0.03
**Auditory**	13(13)	7(7)	χ ^2^=2.00 P=0.15
**Respiratory**	20(2)	2(2)	χ ^2^=16.55 P≤0.001
**Cardiovascular**	33(33)	12(12)	χ ^2^=11.54 P=0.001
**Musculoskeletal**	60(60)	36(36)	χ ^2^=12.66 P≤0.001
**Neurological**	47(47)	17(17)	χ ^2^=10.67 P=0.001
**Dermatological**	12(12)	6(6)	χ ^2^=2.19 P=0.14

Table 3 shows that the prevalence of obesity, hypertension, dyslipidemia and psychological distress are statistically significantly higher among train drivers compared to the comparison group (P≤0.001).

**Table 3.** Morbidity pattern of the study groups.

Morbidity pattern	Train drivers n=100	Comparison group n=100	Test of significance and P value
	N (%)	N (%)	
**Obesity**	62(62)	12(12)	χ ^2^=53.63, P≤0.001
**Hypertension** ^**b**^	46(46)	16(16)	χ ^2^=21.03, P≤0.001
**Dyslipidemia** ^**c**^	67(72.8)	33(35.5)	χ ^2^=25.97, P≤0.001
**Psychological distress**	71(71)	27(27)	χ ^2^=38.74, P≤0.001

^b^ Hypertension cases (46 vs. 16) = previously diagnosed cases of HTN (26 vs. 11) - in addition to newly discovered cases of HTN (20 vs. 5).

^c^ Participants who accepted to give a blood sample for laboratory investigation - train drivers (n=92) & comparison group (n=93).

Table 4 shows that there is a higher prevalence of presenteeism among train drivers compared to the comparison group (76% and 31%, respectively) with a highly statistically significant difference (P≤0.001). In total, 54 persons out of 76 (71.1%) train drivers with presenteeism have lower scores (≤18) of the Stanford Presenteeism Scale and reduced performance at work compared to 13 persons out of 31 (41.9%) among the comparison group. The mean score of SPS-6 is significantly (P≤0.001) lower among train drivers (15.7±3.7) compared to the comparison group (19.2±2.9).

**Table 4.** Presenteeism among study groups measured by Stanford Presenteeism Scale (SPS-6).

Presenteeism	Train drivers n=100	Comparison group n=100	Test of significance and P value
	N (%)	N (%)	
**Presenteeism**			
Present	76(76)	31(31)	χ ^2^=40.7 P≤0.001
Absent	24(24)	69(69)	
**SPS-6 score** ^d^			
≤18	54(71.1)	13(41.9)	χ ^2^=7.98 P=0.005
>18	22(28.9)	18(58.1)	
**Mean ±SD**	15.7 **±**3.7	19.2±2.9	t=4.8, P≤0.001

^d^ Percentage within presenteeism. SD=standard deviation.

The bivariate analysis ( Table 5) shows that all participants (100%) with psychological distress reported presenteeism. Furthermore, logistic regression analysis shows that being a train driver (AOR=5.4) and having hypertension (AOR=4.03) are independently associated with the likelihood of having presenteeism.

**Table 5.** Logistic regression analysis of independent predictors of presenteeism among study groups.

Risk factors	Total	Presenteeism N (%)	COR (95%CI)	Logistic regression
	200	107(53.5)		AOR (95%CI)
**Study group**				
Train drivers	100	76(76)	7.05(3.8-13.2) ***	5.4(2.8-10.4)
Comparison group	100	31(31)	(r)	(r)
**Shift type**				
Day	115	43(37.4)	(r)	
Night/alternating	85	64(75.3)	5.1(2.7-9.5) ***	
**Obesity**				
Obese	74	51(68.9)	2.8(1.5-5.1) **	
Non obese	126	56(44.4)	(r)	
**Hypertension**				
Yes	62	50(80.6)	5.9(2.9-12.1) ***	4.03(1.9-8.7)
No	138	57(41.3)	(r)	(r)
**Dyslipidemia** ^**e**^				
Yes	100	69(69)	4.3(2.3-7.9) ***	
No	85	29(34.1)	(r)	
**Psychological distress**				
Yes	98	98(100)	Unlimited ***	
No	102	9(8.8)	(r)	
**Musculoskeletal symptoms**				
Yes	96	61(63.5)	2.2(1.2-3.9) **	
No	104	46(44.2)	(r)	
**Cardiovascular symptoms**				
Yes	45	34(75.6)	3.5(1.6-7.3) ***	
No	155	73(47.1)	(r)	
**Neurological symptoms**				
Yes	50	33(66)	1.9(1.02-3.9) *	
No	155	74(49.3)	(r)	

*, ** and *** = significant difference at P≤0.05, P≤0.01 and P≤0.001 respectively.

^e^ Total for dyslipidemia =185 (15 subjects are missed).

## Discussion

Presenteeism is a term used when employees come into work despite physical or psychological health problems. So, they may not be able to fully perform their duties and are more likely to make mistakes on their job. In the present study, almost all physical complaints and morbidities were more frequent among train drivers compared to the comparison group with a statistically significant difference (P≤0.05) ( Tables 2, 3). Train driving is a high-level job where the workers’ ill health may lead directly to a serious incident affecting the rail network and public safety since, the vigilance and attention of train drivers are crucial to their job. They are also responsible for people’s lives. So, going to work despite physical or psychological health problems (presenteeism) may increase the risk of occupational injuries and train accidents.

The number of existing studies on presenteeism among train drivers is scant, and most studies on presenteeism have analyzed healthcare workers especially nurses. ^33^^–^^36^

In the current study, there is a higher prevalence of presenteeism among train drivers (76%) compared to the comparison group (31%) with a high statistically significant difference (P≤0.001) ( Table 4). Logistic regression analysis shows that being a train driver (AOR=5.4) is an independent predictor of presenteeism ( Table 5).

The prevalence of presenteeism among Egyptian train drivers in the current study (76%) is shown to be similar to that of nurses (76.2%) working in hospitals of Croatia. ^34^ However, a lower prevalence of presenteeism (52%) was detected among railroad workers in Korea. ^12^ Also, a lower prevalence was detected in other occupations such as police officers in Sweden (46.5%), ^37^ workers at a food industrial company in Brazil (50.9%) ^5^ and employees in South Korea (41.2%). ^38^

Presenteeism is usually common among workers whose occupations involve high job demands and relatively large individual responsibility, where the personnel are required to be in place, with minimal chance for temporary replacement such as; train drivers and health care providers. In such occupations, inadequate physical and psychological status of the affected workers can interfere with maintaining vigilance and concentration. ^35^^,^^37^ Presenteeism is common among train drivers than other railway occupations due to higher job strain among train drivers. ^12^

In the present work, bivariate analysis shows that all participants (100%) with psychological distress reported presenteeism ( Table 5). Similarly, several studies support the positive association between psychological distress and presenteeism. ^11^^,^^39^^–^^41^ Psychological health problems may be more linked to presenteeism than absenteeism because it may be more difficult to ensure that absence is due to this reason. ^2^

Train drivers were exposed to several psychosocial risk factors in the workplace affecting their mental and psychological wellbeing and may result in mental and psychological health problems, such as; shift work, high job demands, limited decisional latitude and job insecurity which can adversely affect their health and directly influence the prevalence of presenteeism. ^14^^–^^16^ Working night and/or alternating day and night shifts was shown to be associated with presenteeism in our study in bivariate analysis ( Table 5). This was compliant with a study conducted upon Korean workers in which a higher presenteeism was reported among shift workers than non-shift workers. ^42^ Shift workers are particularly vulnerable to long hours of duty and insufficient rest elevating their risk to develop presenteeism.

The current study revealed a positive association between different health conditions and presenteeism, logistic regression analysis shows that hypertension was an independent predictor for presenteeism (AOR=4.03) ( Table 5). This was in agreement with a study conducted among Chinese workers where a higher prevalence of presenteeism was found among workers with high blood pressure. ^20^ Similarly in the United States, all individuals with hypertension were more likely to report lost productive time (LPT) while at work (presenteeism) compared to normotensive individuals. ^43^ However, there was no significant association between lost productivity and hypertension in a study conducted to assess the effect of different cardio-metabolic risk factors including hypertension on productivity. ^44^ The greater impact of hypertension on LPT and presenteeism can be explained by hypertension being largely undertreated despite its high prevalence rates, ^45^ probably due to late access to health care and poor compliance to medication regimens resulting in inadequate control of hypertension, so, the workers may go to work while ill (hypertensive). ^46^^,^^47^

A significant association between presenteeism and obesity was detected in bivariate analysis ( Table 5) which was similarly found in workers in Petrochemical industry in China ^20^ and workers at a food industrial company in Brazil. ^5^ This could be attributed to sedentary work of train drivers and its negative impact on their health. Sedentary work with insufficient movement and muscle activity, low energy expenditure and lack of changes in posture may result in low physical activity and obesity. ^48^ Also, the high job demands among train drivers can cause stress and unhealthy dietary behaviors that may result in obesity and increase its negative impact on health and consequently greater adverse workplace effects such as presenteeism. ^49^ Furthermore, obesity is considered an important risk factor for cardiovascular disease, as it can increase the prevalence and severity of cardiovascular risk factors such as; diabetes mellitus (type II), dyslipidemia and hypertension. ^50^ So, it can significantly exacerbate the adverse effects of these conditions on productivity. ^44^

Moreover, the present findings revealed a significant association between musculoskeletal complaints and presenteeism among train drivers ( Table 5). Correspondingly in Brazil, a positive association was found between presenteeism and occurrence of musculoskeletal symptoms. ^5^ Musculoskeletal problems may interfere with work and daily life activities as a result of functional limitations. They also arouse feelings of ineffectiveness and uselessness resulting in a lack of productivity. ^51^ So, targeting and assessment of the underlying health risks that might lead to presenteeism in the workplace is a critical issue for its control and management.

## Conclusions

In the present study, the prevalence of presenteeism and its associated risk factors are significantly higher among train drivers than the comparison group. All participants with psychological distress reported presenteeism. Being a train driver and having hypertension are independently associated with the likelihood of having presenteeism. There is an urgent need for the railway industry to understand the factors that contribute to presenteeism. Of particular interest are, the use of effective health promotion programs and effective physical and psychological assessment that may play a role in increasing worker productivity and reduction in presenteeism. Provision of enough rest periods after shifts, and regulation of work to facilitate sick leaves when needed are recommended to ameliorate presenteeism. A large scale national study including all train drivers is recommended.

### Study limitations

The study was conducted in single locality with a relatively small sample size. So, the results can’t be generalized to all train drivers. There is a possibility of recall bias in physical complaints such as; musculoskeletal, cardiovascular and neurological complaints.

## Data availability

### Underlying data

Harvard Dataverse: Presenteeism and associated factors among railway train drivers. https://doi.org/10.7910/DVN/CG8Z1K. ^52^

### Extended data

Harvard Dataverse: questionnaire and informed consent form for “Presenteeism and associated factors among railway train drivers”. https://doi.org/10.7910/DVN/ZGY5UB. ^53^

This project contains the following extended data:

- informed written consent.doc
- questionnaire.docx

Data are available under the terms of the Creative Commons Zero “No rights reserved” data waiver (CC0 1.0 Public domain dedication).
