# Supplementary material for: Presenteeism and associated factors among railway train drivers
Source: F1000Res. 2022 Jun 1;11:470. Originally published 2022 Apr 28. [Version 2] doi: 10.12688/f1000research.111999.2 (PMC9237557; doi:10.12688/f1000research.111999.2)
Supplement: Peer review history

# Peer review: Reviewer response for version 2

**DOI:** 10.5256/f1000research.134451.r139552

**Published:** 2022-06-27

**Authors:** Alisha McGregor

**Affiliations:** 1 School of Psychology, University of Wollongong, Wollongong, NSW, Australia

I have looked at the authors revisions and are happy to endorse this article.

Is the work clearly and accurately presented and does it cite the current literature?

Yes

If applicable, is the statistical analysis and its interpretation appropriate?

Yes

Are all the source data underlying the results available to ensure full reproducibility?

No source data required

Is the study design appropriate and is the work technically sound?

Partly

Are the conclusions drawn adequately supported by the results?

Yes

Are sufficient details of methods and analysis provided to allow replication by others?

Yes

Reviewer Expertise:

NA

I confirm that I have read this submission and believe that I have an appropriate level of expertise to confirm that it is of an acceptable scientific standard.

# Peer review: Reviewer response for version 2

**DOI:** 10.5256/f1000research.134451.r140098

**Published:** 2022-06-21

**Authors:** Amal ElSafty

**Affiliations:** 1 Department of Occupational and Environmental Medicine, Faculty of Medicine, Cairo University, Giza, Egypt

This is an interesting study and generally well written and structured. The authors have collected data from all working population so results can be fairly extrapolated. It can be also of great importance, especially with the recent increase of train accidents in Egypt.

 Below I have provided few remarks, and other than that, from my  point of view it is accepted for indexing.

1. Comments on Table 2 in the results, ignore the respiratory complaints and no discussion on this item.
2. 2 ^nd^ sentence in the first paragraph in the discussion studied the impact of presenteeism on work performance, however this manuscript is investigating the effect of presenteeism on train driver’s health. This sentence can be used to suggest a warning sign for probable accidents because of high presentism among the studied train drivers.
3. 8 ^th^ paragraph in discussion demonstrated the relation between presentism and hypertension. The second half of this paragraph mostly explains hypertension with lost productive time (LPT), which is not studied in this research.
4. Recommendations might also include the importance of occupational psychologist to combat stressors and proper.

Is the work clearly and accurately presented and does it cite the current literature?

Yes

If applicable, is the statistical analysis and its interpretation appropriate?

Yes

Are all the source data underlying the results available to ensure full reproducibility?

Yes

Is the study design appropriate and is the work technically sound?

Yes

Are the conclusions drawn adequately supported by the results?

Yes

Are sufficient details of methods and analysis provided to allow replication by others?

Yes

Reviewer Expertise:

Occupational  and Environmental Medicine

I confirm that I have read this submission and believe that I have an appropriate level of expertise to confirm that it is of an acceptable scientific standard.

# Peer review: Reviewer response for version 1

**DOI:** 10.5256/f1000research.123725.r136241

**Published:** 2022-05-25

**Authors:** Alisha McGregor

**Affiliations:** 1 School of Psychology, University of Wollongong, Wollongong, NSW, Australia

Thanks for giving me the opportunity to review this article on presenteeism amongst train drivers in Egypt. 

 I have some comments for consideration below. 

- In the intro, the author says that presenteeism results in greater productivity losses than absenteeism, which is correct over the long term; However, on any given day that a worker chooses presenteeism over absenteeism they are going to be more productive. In that even if they are only 20% productive when they come into work ill this is still more than if they chose to take the day off (i.e., 0% productivity). I think this should be considered when talking about productivity losses associated with presenteeism compared to absenteeism. 
- The author has used Aronsson and Gustafsson definition of presenteeism in the intro but then measures the construct using the SPS-6 scale which incorporates productivity into the measure. The author could consider modifying their definition of the construct to be consistent with the way they have measured presenteeism in the study.
- Very limited discussion of the predictors of presenteeism in the intro, this could be expanded upon.
- In the methods section - the use of the heading 'flow of work' seems odd. I have never seen the study procedure described in this way. Please review.

Is the work clearly and accurately presented and does it cite the current literature?

Yes

If applicable, is the statistical analysis and its interpretation appropriate?

Yes

Are all the source data underlying the results available to ensure full reproducibility?

No source data required

Is the study design appropriate and is the work technically sound?

Partly

Are the conclusions drawn adequately supported by the results?

Yes

Are sufficient details of methods and analysis provided to allow replication by others?

Yes

Reviewer Expertise:

Presenteeism

I confirm that I have read this submission and believe that I have an appropriate level of expertise to confirm that it is of an acceptable scientific standard, however I have significant reservations, as outlined above.

# Response

**Published:** 2022-05-25

**Authors:** Asmaa Awaad

**Affiliations:** Mansoura University, Egypt

We thank Dr. McGregor for reviewing our manuscript. We present our responses below each comment as well as a new version of our manuscript (Version #2)

 In the intro, the author says that presenteeism results in greater productivity losses than absenteeism, which is correct over the long term; However, on any given day that a worker chooses presenteeism over absenteeism they are going to be more productive. In that even if they are only 20% productive when they come into work ill this is still more than if they chose to take the day off (i.e., 0% productivity). I think this should be considered when talking about productivity losses associated with presenteeism compared to absenteeism. 

**Response:** Thank you, the term "over the long term" is added to the sentence (presenteeism involves higher productivity losses than absenteeism over the long term)

 The author has used Aronsson and Gustafsson definition of presenteeism in the intro but then measures the construct using the SPS-6 scale which incorporates productivity into the measure. The author could consider modifying their definition of the construct to be consistent with the way they have measured presenteeism in the study.

**Response:** Thank you, the definition is modified to be consistent with the way we have measured presenteeism in the study.

 Very limited discussion of the predictors of presenteeism in the intro, this could be expanded upon.

**Response:** Thank you, pain including; musculoskeletal and neurological pain are added to the predictors of presenteeism in the intro.

 In the methods section - the use of the heading 'flow of work' seems odd. I have never seen the study procedure described in this way. Please review.

**Response:** Thank you, the heading 'flow of work' is replaced by "study procedure"
